# Piloting a complex intervention to promote a tobacco and alcohol-free pregnancy: the Smoke and Alcohol Free with EHealth and Rewards (SAFER) pregnancy study

**DOI:** 10.1186/s12884-022-05320-8

**Published:** 2023-01-10

**Authors:** Leonieke J. Breunis, Marlou L. A. de Kroon, Lieke C. de Jong-Potjer, Eric A. P. Steegers, Jasper V. Been

**Affiliations:** 1 Department of Obstetrics and Gynaecology, Division of Obstetrics and Fetal Medicine, Erasmus MC Sophia Children’s Hospital, University Medical Centre Rotterdam, Rotterdam, The Netherlands; 2 Department of Health Sciences, University Medical Center Groningen, University of Groningen, Groningen, The Netherlands; 3 Department of Public Health and Primary Care, Environment and Health, KU Leuven, Leuven, Belgium; 4 Department of Paediatrics, Division of Neonatology, Erasmus MC Sophia Children’s Hospital, University Medical Centre Rotterdam, room SK-3216, Wytemaweg 80, Rotterdam, CN 3015 The Netherlands; 5 Department of Public Health, Erasmus MC, University Medical Centre Rotterdam, Rotterdam, The Netherlands

**Keywords:** Smoking cessation, Alcohol cessation, Pregnancy, Preconception, Incentives, Group sessions, eHealth, Web-based platform

## Abstract

**Background:**

Tobacco smoking and alcohol consumption before and during pregnancy increase the risk of adverse health outcomes for mother and child. Interventions to address smoking and drinking before and during pregnancy have the potential to reduce early-life health inequalities. In the Smoke and Alcohol Free with EHealth and Rewards (SAFER) pilot study we aimed to evaluate the acceptability, feasibility and effectiveness of a complex intervention supporting women in smoking and alcohol cessation before and during pregnancy.

**Methods:**

From February 2019 till March 2021, we piloted the SAFER pregnancy intervention among pregnant women and women planning pregnancy in South-West Netherlands in an uncontrolled before-after study. Participants were supported in smoking and alcohol cessation via up to six group sessions and an online platform. In addition, biochemically validated cessation was rewarded with incentives (i.e. shopping vouchers) amounting up to 185 euros. We aimed to include 66 women. The primary outcome was smoking and/or alcohol cessation at 34–38 weeks of gestation (if pregnant) or after six group sessions (if not pregnant). Quantitative data were analysed using descriptive statistics. Focus group interviews among those involved in the study were conducted at the end of the study to explore their experiences. Qualitative data was analysed using thematic analysis.

**Results:**

Thirty-nine women who smoked were included; no women who consumed alcohol were referred to the study. Unemployment (51%), financial problems (36%) and a smoking partner (72%) were common. Thirteen women (33%) dropped out, often due to other problems impeding smoking cessation or ‘being too busy’ to participate in the group sessions. Eleven women (28%) had quit smoking at the study’s endpoint. The personal and positive approach was highly valued and biochemical validation was felt to be helpful.

**Conclusion:**

The SAFER pregnancy intervention seems appropriate for women in need of extra support for smoking cessation before and during pregnancy. Its impact on alcohol cessation could not be studied due to recruitment issues. Recruitment and prevention of early dropout need attention in further development of this intervention.

**Trial registration:**

Netherlands Trial Register: NL7493. Date registered: 04/02/2019.

**Supplementary Information:**

The online version contains supplementary material available at 10.1186/s12884-022-05320-8.

## Introduction

Smoking decreases fertility, and during pregnancy it increases the risk of miscarriage and many adverse perinatal outcomes, such as preterm delivery, birth defects and perinatal death [1–3]. Children born to mothers who smoke are also more likely to develop asthma, respiratory infections, obesity and to take up smoking [4–6]. Alcohol consumption during pregnancy is associated with miscarriages, fetal growth restriction, preterm delivery and Fetal Alcohol Spectrum Disorders [7, 8]. In Europe, one in seventeen pregnant women was a daily smoker in 2015 and one in four women consumed alcohol during pregnancy (2012) [9, 10]. In the Netherlands, contemporary data indicate that 4% of women smoke during their entire pregnancy. Although 4% of pregnant women reported alcohol consumption at any time during pregnancy, screening data indicate that this is an underestimation [11, 12].

Only half of women who smoke, successfully quit smoking during pregnancy [9]. Women who have an unplanned pregnancy, a partner who smokes, financial problems, and a low socioeconomic status (SES) less often successfully quit smoking during pregnancy [13, 14]. Young maternal age and frequent alcohol use before pregnancy are risk factors for sustained alcohol use during pregnancy [15–17]. Importantly, smoking and alcohol use during pregnancy often co-occur [17, 18].

Although interventions to promote smoking cessation before and during pregnancy, such as health education, counselling and pharmacological support, can be effective among those unable to quit themselves, the vast majority continues smoking despite such interventions [19, 20]. Interventions for alcohol abstinence particularly focused on pregnant women and women planning pregnancy are scarce [21]. Development of more effective interventions tailored at these women who continue to smoke or use alcohol is needed.

Knowledge about the dangers of smoking and alcohol use, social support and health literacy are important facilitators of smoking and alcohol cessation during pregnancy [19, 22, 23]. eHealth-based interventions can also be effective. A recent meta-analysis showed a relative risk (RR) for smoking cessation of 3.06 [95% confidence interval (CI) 1.28–7.33] and the odds ratio (OR) for alcohol cessation varies between 2.77 and 4.72 among studies in favour of the eHealth-based intervention [24–26]. Financial incentives (i.e. rewards for a specific goal with the purpose to motivate) are effective in promoting smoking cessation during pregnancy (RR 2.38 [95% CI 1.54–3.69]) [27, 28] and may also help reduce alcohol use, although evidence on the latter is inconclusive [29–31]. A combination of various existing interventions might enhance effectiveness compared to a single intervention, but this has not been extensively tested to support smoking and alcohol cessation before and during pregnancy [19, 20].

In the Smoke and Alcohol Free with EHealth and Rewards (SAFER) pregnancy study, we piloted a complex intervention consisting of a combination of group sessions (to increase knowledge, social support, and health literacy), access to a web-based platform with customised health information, and provision of financial incentives to promote cessation of smoking and alcohol use in pregnant women and women with a wish to conceive. We aimed to assess acceptability, feasibility, and effectiveness of the intervention to promote smoking and alcohol cessation before and during pregnancy.

## Methods

The SAFER pregnancy study was a prospective, uncontrolled before-after study in primary care, undertaken according to a pre-specified peer-reviewed study protocol (Netherlands Trial Register: NL7493, registered on 4 February 2019) [32]. Since during the study no women were referred for alcohol use, we mainly focus our current Methods description on the approach to women referred for smoking cessation.

### Setting and participants

The study was conducted in the South-West region of the Netherlands. We aimed to include 66 women, with no predefined distribution of pregnancy status [32]. In February 2019, the study started in the municipality of Zoetermeer and Benthuizen. Due to recruitment challenges, this was expanded to parts of Rotterdam (December 2019) and The Hague (March 2020). Women were eligible if they were pregnant or had a wish to become pregnant within six months and smoked at least one cigarette a day. Women were excluded if they were less than 18 years of age, more than 20 weeks pregnant, had insufficient mastery of the Dutch language, were unwilling to undergo urinary and/or exhaled breath testing, used hard drugs, or had a urinary cotinine level below 50 µg/L [33] and/or exhaled carbon monoxide (CO) level less than 7 parts per million (ppm) [34, 35] at inclusion.

Eligible women were informed about the study by their healthcare provider or through promotion material (e.g. posters at schools). If a woman was potentially interested in participation, the healthcare provider or the woman herself sent contact information to the researcher (LB), who contacted the woman via telephone. If she was still interested in participation, a home visit was scheduled.

After the first phone call, information about the study and the informed consent form were sent by e-mail. During the home visit, the study was further explained, the informed consent form was signed, and biochemical validations (urinary cotinine level and CO breath test) were performed. A cessation plan was devised together with the participant. If a participant was interested in using nicotine replacement therapy (NRT) and met the criteria for use [36], she was referred to a healthcare provider with experience with the use of NRT during pregnancy. Additional smoking cessation support as part of routine care initiated by health care providers (e.g. midwife, obstetrician, general practitioner) was allowed.

During the first two waves of the COVID-19 pandemic (March to May 2020 and November 2020 to March 2021) home visits were not possible according to Erasmus MC policy and were temporarily replaced with a second phone call.

### Intervention

Participants received free access to an existing web-based platform: Smarter Pregnancy [37]. The platform determined a risk profile of participants’ lifestyle using online surveys and stimulated a healthy lifestyle using personalised e-mails containing information and advice.

In addition, monthly group sessions were organised in Zoetermeer, Rotterdam and The Hague. The group sessions aimed to provide peer support, increase self-efficacy, stimulate avoiding risk behaviour, and promote adoption of a healthy lifestyle by providing information and organising activities concerning a healthy diet, sport activities, and cessation of smoking and alcohol use. There was no particular sequence of sessions; as such, women could enter the study at any time point. Participants were invited to attend a maximum of six sessions, and participation in the sessions discontinued after the women had given birth. Each session lasted approximately two hours. The first half hour was used to introduce participants to each other and to share experiences in quitting. The next 1.5 h were used to do yoga, do Pilates, receive information on risk of smoking, think about and discuss perceived identity, cook a meal together, knit together, do mindfulness training, receive information about pregnancy and the postpartum period, or do a voice liberation singing session (withdrawn during COVID-19 pandemic) [32]. LB was present at each session, and during some sessions an additional instructor was present.

If a participant had quit smoking, this was biochemically validated using the CO breath test. If the CO breath test reading was below 7 ppm, the participant received a voucher as incentive (monetary value provided in Additional file 1: Table S1). The longer cessation was sustained, the more value the voucher had. The same monetary value was also deposited in a group-based incentive, that was later used for a joint project selected and developed by the participants. During the two COVID-19 waves no biochemical validation was possible and self-reported abstinence was used instead as an indicator of smoking cessation.

### Data collection

The primary outcome was biochemically validated (by CO breath test and urinary cotinine) smoking cessation at:

- week 34 to 38 of gestation; or
- the moment of giving birth if preterm labour occurred; or
- the last validation after six group sessions in those women who did not become pregnant during participation; or
- the end of the project period if not pregnant at the time.

Secondary outcomes were: process variables (study log), perceived barriers and facilitators of implementation (study log and focus group study), perceived efficiency and appreciation of the intervention (questionnaires among participants and focus group study), perceived identity changes (questionnaires among participants), and pregnancy outcomes (questionnaires among participants). Participants received questionnaires at start of participation, a week before each group session, at the primary endpoint and after delivery. The questionnaires were based on a combination of existing questionnaires such as the Fägerstrom questionnaire [38] translated into Dutch and questions developed by our study group. Questions concerned baseline characteristics, level of addiction, obstetric characteristics, identity and opinion on aspects of the SAFER pregnancy intervention. The questionnaires were tested in former participants of the focus group study before the SAFER pregnancy study [32]. Most questions were multiple choice with some open-ended questions for further exploration. A more detailed description of the questionnaires can be found in our protocol [32].

Three semi-structured focus groups were conducted at the end of the study including moderators of the six group sessions, former participants, and involved healthcare providers, respectively. These where led by an experienced moderator and notes were taken by an assistant. Due to the COVID-19 pandemic, all focus groups were conducted online using MS Teams. Each focus group lasted approximately 1.5 h.

### Data analysis

The proportion of women who quit smoking at the primary endpoint was calculated as the number of women who had quit at this endpoint divided by the total number of women included in the study. In this analysis, women who discontinued participating or who were lost to follow-up were conservatively considered not to have quit smoking. Descriptive statistics are used for the secondary outcomes and results are reported in narrative and tabular form. Audio recordings of the focus group study were transcribed verbatim. Transcripts were coded and divided into main themes (thematic content analysis) by one of the researchers (LB). To minimise interpreter bias, the focus group leader (VM) independently validated the conclusions.

## Results

### Process outcomes

**Fig. 1:**
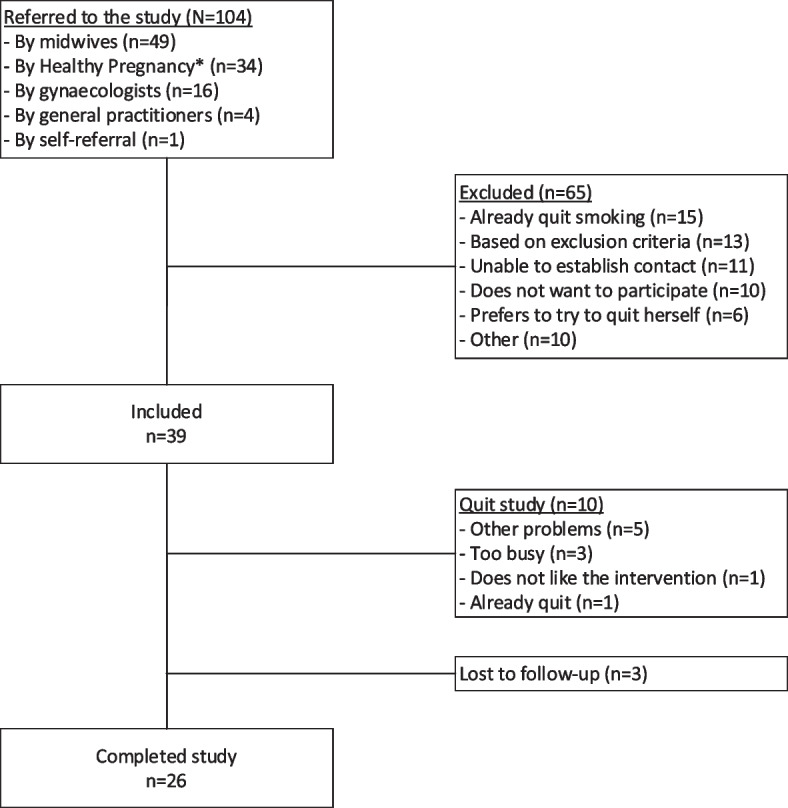
Study population

One hundred four women were referred to the study, mostly by their midwife (47%; Fig. 1). 65 women (63%) were excluded, most often because they had already quit smoking (*n* = 15) or based on the exclusion criteria (*n* = 13). 39 women were included in the SAFER pregnancy study, all because of smoking; none of the women fulfilled the criteria for inclusion based on alcohol use.

### Study population

Most participants lived in rental houses (67%) and were unemployed (51%), see Table 1. One-third of participants had financial problems. 32 women (82%) were pregnant at inclusion, of whom 19 (59%) had an unplanned pregnancy. Median gestational age at inclusion was 12 weeks (IQR 10–15). At start of the study, 45% of women had a Fagerström score of five or higher (indicating significant nicotine dependence [38]) and 59% had the intention to quit smoking within one month (according to the Motivation To Stop Scale (MTSS); Table 2) [39]. Most women had a partner who also smoked (72%).

**Table 1:** Characteristics of the study population^a^

Characteristic	Category	Abstinent at primary endpoint (*n* = 11)	Current smoker at primary endpoint (*n* = 15)	Stopped participation or lost-to-follow-up (*n* = 13)
**Age**	Median (IQR)	35 (27–38)	31 (29–38)	30 (27–32)
**Country of birth**	The Netherlands	10 (91)	14 (93)	10 (77)
	Netherlands Antilles	0 (0)	0 (0)	1 (8)
	Germany	0 (0)	1 (7)	1 (8)
	Iraq	0 (0)	0 (0)	1 (8)
	United States	1 (9)	0 (0)	0 (0)
**Marital status**	Married or registered partnership	2 (18)	3 (20)	1 (8)
	Living together	5 (46)	7 (47)	4 (31)
	Committed relationship, not living together	2 (18)	5 (33)	6 (46)
	Single	2 (18)	0 (0)	2 (15)
**Living situation**	Rental home	9 (82)	10 (67)	7 (54)
	House owner	2 (18)	2 (13)	3 (23)
	Living with parents/family/friends	0 (0)	3 (20)	1 (8)
	Assisted living	0 (0)	0 (0)	1 (8)
	No permanent residence	0 (0)	0 (0)	1 (8)
**Education level**^**b**^	Low (special education, elementary school, high school)	1 (9)	2 (13)	6 (46)
	Middle (Secondary vocational education)	5 (46)	12 (80)	6 (46)
	High (University of applied sciences, university)	5 (46)	1 (7)	1 (8)
**Employment**	Yes, fulltime	4 (36)	3 (20)	3 (23)
	Yes, part-time	3 (27)	2 (13)	3 (23)
	Unemployed	4 (36)	9 (60)	7 (54)
	Missing	0 (0)	1 (7)	0 (0)
**Net family income**	< 1000 euro/month	0 (0)	2 (13)	2 (15)
	1000–1499 euro/month	2 (18)	2 (13)	4 (31)
	1500–1999 euro/month	0 (0)	4 (27)	2 (15)
	2000–2499 euro/month	2 (18)	2 (13)	2 (15)
	2500–2999 euro/month	2 (18)	4 (27)	1 (8)
	> 3000 euro/month	5 (46)	1 (7)	2 (15)
**Self-reported financial problems**	Yes	3 (27)	4 (27)	7 (54)
	No	8 (73)	11 (73)	6 (46)
**Self-reported relational problems (partner or family)**	Yes	7 (64)	0 (0)	2 (15)
	No	4 (36)	15 (100)	11 (85)
**Drug use**	No	9 (82)	12 (80)	12 (92)
	Former	1 (9)	3 (20)	1 (8)
	Yes, marihuana	1 (9)	0 (0)	0 (0)
**Alcohol consumption**	Never	3 (27)	4 (27)	2 (15)
	Quit because of pregnancy	5 (46)	5 (33)	7 (54)
	Quit because of other reason than pregnancy	2 (18)	5 (33)	2 (15)
	Yes	1 (9)	1 (7)	2 (15)
**BMI**	Underweight (BMI < 18.5)	1 (9)	0 (0)	0 (0)
	Normal (BMI 18.5–25)	4 (36)	3 (20)	5 (39)
	Overweight (BMI 25–30)	2 (18)	6 (40)	5 (39)
	Moderately obese (BMI 30–35)	3 (27)	4 (27)	2 (15)
	Severely obese (BMI 35–40)	1 (9)	0 (0)	1 (8)
	Very severely obese BMI > 40)	0 (0)	2 (13)	0 (0)

^a^Data provided as n (%), unless stated otherwise

^b^Based on based on the International Standard Classification of Education 2013; except secondary vocational education 1 was classified as middle instead of low

**Table 2:** Characteristics related to smoking of participants at intake^a^

Characteristic	Categories	Abstinent at primary endpoint (*n* = 11)	Current smoker at primary endpoint (*n* = 15)	Stopped participation or lost-to-follow-up (*n* = 13)^b^
**Fagerström score**	Score below 5 (low dependency)	8 (73)	5 (33)	8 (62)
	Score of 5 or higher	3 (27)	10 (67)	4 (31)
**Number of cigarettes smoked per day**	1–4 cigarettes	0 (0)	2 (13)	3 (23)
	5–9 cigarettes	4 (36)	3 (20)	4 (31)
	10–14 cigarettes	4 (36)	3 (20)	5 (39)
	15–19 cigarettes	2 (18)	3 (20)	0 (0)
	20–30 cigarettes	1 (9)	3 (30)	0 (0)
	Missing	0 (0)	1 (7)	1 (8)
**Motivation to quit Score**	I think I should quit, but do not really want to	0 (0)	0 (0)	1 (8)
	I want to quit, but have not thought about when	0 (0)	0 (0)	1 (8)
	I want to quit, but do not know when I will	0 (0)	0 (0)	1 (8)
	I want to quit and hope to do so soon	1 (9)	7 (47)	1 (8)
	I really want to quit and plan on doing this within 3 months	1 (9)	1 (7)	0 (0)
	I really want to quit and plan on doing so in the upcoming month	9 (82)	7 (47)	7 (54)
	Missing	0 (0)	0 (0)	2 (15)
**Age started smoking (years)**	Median (IQR)	15 (13–18)	14 (13–15)	16 (14–17)
**Number of previous quit attempts**	Never	5 (45)	6 (40)	5 (38)
	Once	2 (18)	2 (13)	4 (31)
	More than once	4 (36)	7 (47)	3 (23)
**Total duration of previous smoking cessation**	Never quit	5 (45)	6 (40)	5 (38)
	Less than 1 month	1 (9)	2 (13)	1 (8)
	1–6 months	1 (9)	4 (27)	4 (31)
	7–12 months	1 (9)	1 (7)	2 (15)
	13–24 months	1 (9)	2 (13)	0 (0)
	25–48 months	2 (18)	0 (0)	0 (0)
**Partner who smokes**	No partner	2 (18)	0 (0)	2 (15)
	No	1 (9)	3 (20)	2 (15)
	Yes	8 (73)	12 (80)	8 (67)
**How often in an environment with cigarette smoke**	Less than once a month	1 (9)	0 (0)	0 (0)
	More than once a month-less than once a week	2 (18)	2 (13)	1 (8)
	1–6 days a week	3 (27)	3 (20)	3 (23)
	Daily	5 (46)	9 (60)	8 (62)
	Missing	0 (0)	1 (7)	1 (8)

^a^Data provided as m (%), unless stated otherwise

^b^One participant did not fill in these questions, percentages based on total number of participants in this group (*n* = 13)

Of the 39 included women, ten (26%) stopped participating prematurely (Fig. 1), most often due to having other problems hindering smoking cessation (*n* = 5; 13%) or ‘being too busy’ to participate in the group sessions (*n* = 3; 8%). Three women (8%) were lost to follow-up. Women who stopped participating or were lost to follow-up (‘dropped-out’) were more likely to live without their partner, have lower educational level, have financial problems, and be less motivated to stop smoking based on the MTSS [39].

### Smoking cessation

**Fig. 2:**
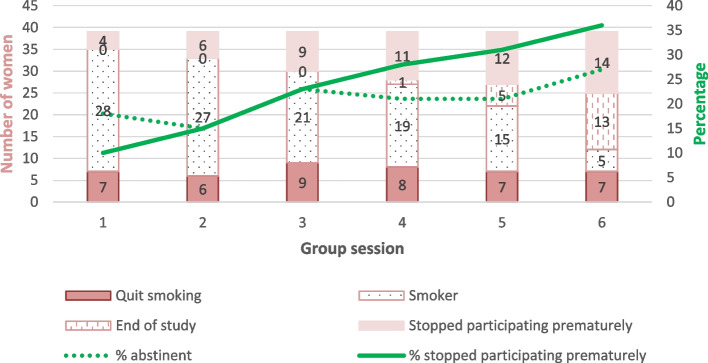
Point prevalence of smoking abstinence and lost-to-follow-up per group session

At the study endpoint, eleven women (28% of those included; 42% of those who finished the study) were abstinent (eight biochemically verified, three self-reported due to COVID-19; nine pregnant women and two planning pregnancy). All women who were abstinent at the primary endpoint had a MTSS of 4 or higher (willing to quit smoking soon; Table 2). Figure 2 shows the number of women who had quit smoking, still smoked, dropped out, and already finished the study (because they had given birth before attending six group sessions) at each group session and the percentage of women who were abstinent or dropped out. Of the twenty pregnant women who completed the study, nine (47%) were abstinent at 34–38 weeks of pregnancy and eight (40%) still were in the first week after giving birth (Table 3). In the questionnaire at the study endpoint, most women who had quit smoking had an intention to remain abstinent (see Additional file 2: Table S2; *n* = 7, 64%).

**Table 3:** Pregnancy and neonatal outcomes^a^

Characteristic	Category	Abstinent at primary endpoint (*n* = 9)	Not abstinent at primary endpoint (*n* = 11)
**Place of delivery**	Home	0 (0)	1 (9)
	Primary care birth centre	0 (0)	0 (0)
	Hospital with midwife	2 (22)	1 (9)
	Hospital with obstetrician	7 (78)	9 (82)
**Type of delivery**	Vaginal	5 (56)	7 (64)
	Instrumental vaginal	1 (11)	1 (9)
	Caesarean section	2 (22)	3 (27)
**Gestational age at delivery**	< 32 weeks	0 (0)	1 (9)
	32–36 weeks	1 (11)	1 (9)
	> 36 weeks	8 (89)	9 (82)
**Sex of the neonate**	Male	4 (44)	6 (55)
	Female	5 (56)	5 (46)
**Median birth weight (IQR)**		3340 (2890–3760)	2845 (2448–3506)
**Apgar score at 5 min**	Below 7	0 (0)	0 (0)
	7 or above	4 (44)	3 (27)
	Missing	5 (56)	8 (73)
**Admitted to hospital after birth**	Yes, paediatric department	2 (22)	1 (9)
	Yes, intensive care unit	0 (0)	2 (18)
	No	7 (78)	8 (73)
**Birth defect**	Yes	1 (11)^b^	0 (0)
	No	8 (89)	11 (100)
**Breast feeding one week postpartum**	Completely	3 (33)	4 (36)
	Partly	3 (33)	1 (9)
	Formula-fed	3 (33)	6 (55)
**Smoking abstinence one week postpartum**	Yes	8 (89)	0 (0)
	No	1 (11)	11 (100)

^a^Data provided as n (%), unless stated otherwise

^b^Club foot (familial)

### Use and appreciation of the SAFER pregnancy intervention

Table 4 shows the use and appreciation of each component of the SAFER pregnancy intervention, according to whether women were abstinent or non-abstinent at the primary endpoint or dropped out.

**Table 4:** Use of the SAFER pregnancy intervention^a^

Component	Category	Abstinent at primary endpoint (*n* = 11)	Not abstinent at primary endpoint (*n* = 15)	Lost-to-follow-up (*n* = 13)
**Care as usual**	Nicotine patches	4 (36)	5 (33)	1(8)
	Nicotine gum	0 (0)	2 (13)	0 (0)
	Varenicline	0 (0)	1 (7)	0 (0)
	E-cigarette	2 (18)	1 (7)	0 (0)
	Quit-smoking coach	2 (18)	4 (27)	2 (15)
**Group sessions**	Median number of invitations for group sessions (IQR)	6 (4–6)	5 (5–6)	3 (1–4)
	Median number of group sessions attended (IQR)	6 (3–6)	3 (2–4)	0 (0–1)
	Median number of online group sessions attended (IQR)	0 (0–1)	1 (1–2)	0 (0–0)
	Only attended live group sessions	5 (45)	3 (20)	3 (23)
	Group sessions helped in smoking cessation^b^	4 (3–5)	3 (3–4)	n.a
	Group sessions helped in remaining abstinent^b^	4 (3–5)	3 (2–4)	n.a
**Online platform**	Accessed platform	6 (55)	3 (20)	3 (23)
	Platform helped in smoking cessation^b,c^	3 (1.5–4)	2 (n.a.)	n.a
**Incentives**	Median monetary value in euros for filling out questionnaires provided per participant	120 (105–135)	120 (90–120)	0 (0–0)
	Median monetary value in euros for smoking cessation provided per participant	55 (35–185)	0 (0–0)	0 (0–0)
	Median times incentives were provided for smoking cessation based on biochemical validation	3 (1–5)	0 (0–0)	0 (0–0)
	Median times incentives were provided for smoking cessation without biochemical validation	0 (0–1)	0 (0–0)	0 (0–0)
	Individual incentives stimulated smoking cessation^b^	4 (2–4)	3.5 (3–4)	n.a
	Individual incentives helped in smoking cessation^b^	3 (2–4)	3 (2–3.5)	n.a
	Group incentives stimulated smoking cessation^b^	3 (2–4)	3 (3–3)	n.a
	Group incentives helped in smoking cessation^b^	3 (2–4)	3 (2–3)	n.a
	Would have participated without incentives	4 (3–4)	3.5 (3–4)	n.a
**Personal contact with the researcher**	Number of participants who initiated contact with the researcher	2 (18)	1 (7)	0 (0)
**Overall study**	Happy I participated^b^	4 (4–5)	4 (4–5)	n.a
	I would recommend participation to others^b^	4 (4–5)	4 (4–5)	n.a
	I made new friends due to participation^b^	3 (3–3)	3 (2–3)	n.a

^a^Data provided as n (%) or median (IQR)

^b^Scores could range from 1 [highly disagree] to 5 [highly agree])

^c^Only those who logged in onto the platform

We planned 33 group sessions in total; eight (24%) were online due to the COVID-19 pandemic. The median invited number of participants per group session was 5 (IQR 4–7), the median number of women who attended was 3 (IQR 2–4). Women who had quit smoking at the primary endpoint attended more group sessions than women who were not abstinent (six [IQR 3–6] versus three [IQR 2–4] group sessions) and attended only live group sessions more often (45%) than women who were not abstinent (20%) or dropped out (23%). Twelve (48%) of the 25 women who filled out the questionnaire at the primary endpoint (highly) agreed that the group sessions helped in smoking cessation. Table 5 shows the appreciation of each group session.

**Table 5:** Appreciation of the group sessions^a^

Subject	Informative (*n* = 15)	Yoga (*n* = 13)	Pilates (*n* = 11)	Cooking (*n* = 5)	Mindfulness (*n* = 9)	Singing (*n* = 4)	Identity (*n* = 20)
Helped in cessation	3 (2–4)	3 (2–4)	3 (2–4)	4 (3–4.5)	3 (2–4)	3 (2.5–3)	3 (2–4)
Helped in living more healthily	3 (2–3)	3 (2.5–3.5)	3 (2–4)	3 (2–3)	3 (2.5–3.5)	2.5 (2–3)	3 (3–3)
Supported in protecting the health of my future baby	4 (2–4)	3 (3–4)	3 (2–4)	3 (2–3.5)	3 (2.5–3.5)	3 (2.5–3)	3 (3–4)
Improved connection with the group	4 (3–4)	3 (3–4)	3 (3–4)	3 (3–4)	3 (3–3.5)	3.5 (3–4.5)	3 (3–4)
I was treated with respect	4 (4–5)	4 (4–5)	4 (4–5)	4 (4–5)	4 (4–5)	4.5 (3.5–5)	4 (4–5)
I received personal attention	4 (4–5)	4 (4–5)	4 (3–5)	4 (4–4.5)	4 (4–4.5)	4 (3.5–4.5)	4 (4–5)
Group session should remain in intervention	4 (4–5)	4 (3–5)	4 (4–5)	4 (4–5)	4 (3–5)	3 (3–3.5)	4 (4–5)
Made me more confident I will be a good mother	3 (3–4)	3 (2–4)	2 (1–3)	3 (1.5–3.5)	2 (2–4)	3 (3–3.5)	3 (2–4)^b^
Made me sad	1 (1–3)	1 (1–3)	1 (1–1)	1 (1–1)	1 (1–1)	2 (1–3.5)	1 (1–2)^b^
Made me angry	1 (1–1)	1 (1–1)	1 (1–2)	1 (1–1)	1 (1–1)	1 (1–2.5)	1 (1–1)^b^
Made me happy	2 (1–3)	4 (1.5–4)	2 (1–4)	3 (3–4)	3 (2–4)	3.5 (3–4)	3 (2–3)^b^
Made me scared	1 (1–2)	1 (1–1)	1 (1–1)	1 (1–1)	1 (1–1)	1.5 (1–3.5)	1 (1–2)^b^
Made me more confident I will succeed in smoking cessation	3 (2–4)	3 (1–4)	1 (1–3)	3 (2.5–4)	3 (2.5–3.5)	3 (3–3.5)	3 (3–4)^b^
Motivated to quit smoking or remain abstinent	3 (2–4)	4 (1.5–4)	3 (1–4)	4 (3–4)	3 (2.5–4)	3.5 (2.5–4)	4 (3–4)

^**a**^Data provided as median (IQR). Answers ranged from highly disagree (1) and highly agree (5)

^b^One missing

Women who quit smoking accessed the online platform more often than women who did not quit smoking (55% versus 20%; Table 4). Women who accessed the platform were neutral in whether the online platform helped in smoking cessation (median appreciation 3 [IQR 2–4]).

Despite many efforts to have local entrepreneurs provide incentives, only five entrepreneurs were willing to provide incentives worth 575 euros. Individual incentives were provided 49 times, representing a total monetary value of 1030 euro, divided over fifteen women. 73% of incentives were provided after biochemical validation, 27% without biochemical validation because of COVID-19. Although 15 of the 25 women who filled out the questionnaire at primary endpoint agreed the personal incentives were stimulating to quit smoking (60%), only seven (28%) felt that the personal incentives had helped in smoking cessation. Women who earned incentives for the group incentive chose to do a group activity, and almost unanimously agreed to also invite the women who did not earn incentives along. Ten women (40% of those who completed the questionnaire) (highly) agreed that the group incentive was stimulating towards smoking cessation.

During participation, most contact between the researcher and participants was via a general smartphone messaging service. Three participants (8%) actively initiated contact with the researcher when support for smoking cessation was needed. However, most supportive contact took place after the researcher initiated contact concerning the planning of the following group session or to remind women to fill out the questionnaire. Contact with the researcher was mostly rated as supportive, although this decreased somewhat towards the end of the intervention (Additional file 3: Fig. S1).

Of the 25 women who filled out the questionnaire at the study endpoint, 21 (84%) were happy that they had participated in this project. No differences were observed between women who were abstinent and not abstinent at the primary endpoint.

### Pregnancy and neonatal outcomes

Table 3 shows the pregnancy outcomes of women who were abstinent and non-abstinent at the primary endpoint. Most children were born full-term (85%) and with a normal birth weight (median birth weight of 3340 (IQR 2890–3760) grams in abstinent women and 2845 (IQR 2448–3506) grams in non-abstinent women).

### Focus group study at the end of the study

All 26 women who had finished the study or stopped participating prematurely were approached. Thirteen agreed to participate, ten were available at the set day and time and seven women showed up. Women who successfully quit smoking (57% vs 28%; see Additional file 4: Table S3) and those who completed the study (100%) were overrepresented. All six group session moderators were invited to a separate focus group and five agreed to participate; although two of them cancelled on the day. All healthcare providers who referred at least one potential participant to the study were approached for a third focus group (*n* = 26). Ten agreed to participate, eight were available on the set date and time and participated. Characteristics of all participants are shown in Additional file 4: Table S3.

Key findings from the focus groups are provided in Table 6. In summary, personal, kind, positive, understanding, and non-judgmental contact with the researcher was very important for the participants to successfully support smoking cessation. In addition, participants found the CO breath test motivating to stop smoking. Participants suggested that more frequent group sessions with group discussions, higher rewards at the start of smoking cessation, and standard provision of NRT could increase effectivity of the intervention. They also felt that online group sessions could be an addition to live group sessions, but should not replace these. The participants felt that online support via the platform was impersonal, and the information provided on the online platform was unfitted and unrealistic. Healthcare providers felt that easy referral was important for success and moderators of the group sessions would prefer more collaboration between all moderators to improve success of the group sessions.

**Table 6:** Results of the focus groups after the SAFER pregnancy study

Subject	Results of the focus groups
Referral to the study and perceived motivation to quit smoking	Pregnant women who smoke are difficult to motivate towards smoking cessation, and midwives fear them leaving their clinic when discussing smoking cessation too often (H) Posters and flyers in the waiting rooms did not help in recruitment, personal conversations between healthcare providers and patients/clients were important (H). Little explanation of the content of the study to patients/clients and easy referral to the researcher were important in the recruitment of participants (H) Participants often have difficulty to quit smoking and remain committed to the study due to psychosocial problems (M), not all healthcare providers agree this complicates smoking cessation (H)
Contact with researcher (including home visit)	Kind, understanding, positive, and non-judgmental approach was important in motivating and supporting participants (P) Initiating contact with the researcher for support when smoking cessation was difficult, was experienced as easy but participants did not often do so (P) The home visit and making of a quit plan were appreciated, however, discussing prevention of complete relapse after lighting up one cigarette was missed (P)
Group sessions	Group sessions were daunting for some pregnant women, demotivating participation (H) One group session per month was too infrequent, more group dialogue about smoking cessation should be an option (P). Two hours per group session was sometimes too long (M) Guiding one or two group sessions per participant was too little to fully help (M) Partners who smoke impede smoke cessation (A), however partners should only partly be involved in the women’s’ intervention (P) Online group sessions can be a good addition, but should not completely replace face-two-face group sessions (P) Collaboration between session moderators could enhance connection between sessions and increase effect and usability for participants (M)
Online platform	Rarely used and if used too many messages were send (P) Platform was impersonal and information was too easy, unfitted for the situation and unrealistic (P)
Incentives	Mixed feelings about motivational effect (P and H), feels like bribery (H) and some participants felt guilty for receiving incentives while others did not receive them despite also doing their best to quit smoking (P) The beginning of smoking cessation is hard, rewards should be highest then (P) Group incentive was not motivational, increases (negative) group pressure (P) Reward for attending group sessions might increase presence (M)
Validation	Breath test was motivating to quit smoking because it very clearly showed the effect of smoking (P). Only one participant was aware that the breath test could already be negative after one day of not smoking (P). Unplanned ‘surprise’ validation would not be appreciated (P)
Investment of time and money	Financial compensation was sufficient (M). Time investment was minimal (H) Participants received less standard care due to the SAFER pregnancy study, as it was felt that the study would replace standard care (H)
Other	Nicotine replacement therapy should be added to the intervention (P)

*P* Participants, *H* Healthcare providers, *M* Moderators of the group sessions, *A* Participants, healthcare providers and moderators of the group sessions

## Discussion

This pilot study indicates that the SAFER pregnancy intervention in addition to standard care is positively valued by participants and may help support smoking cessation before and during pregnancy. At the same time only a limited number of women were referred to the study, women with problematic alcohol use were not reached, and during the study substantial drop-out (33%) occurred. Results from the focus group study afterwards indicated that regular personal contact with someone who is non-judgmental and who remains positive, the biochemical validations and guidance towards existing care such as NRT were facilitators of the SAFER pregnancy intervention.

This is the first study that combined group sessions, a web-based platform and incentives to support women who are pregnant or planning pregnancy in smoking and alcohol cessation. A major strength is that before the development of the study, ethical considerations were explored [40], and the target population was involved in the development using focus groups [32]. We were able to include many women with problems related to a low SES, indicating that the SAFER pregnancy intervention is appealing for women who fail in smoking cessation during pregnancy most often. This is important, as it was recently shown that smoking-related inequalities are growing [41]. Another strength of this study is that we used both quantitative and qualitative approaches to assess acceptability, feasibility and effectiveness of the SAFER pregnancy intervention.

A weakness is that, in view of the pilot stage of our intervention assessment, we pragmatically used a before-after study design without a control group [42, 43]. Inclusion went slowly and we were not able to reach our planned sample size. Moreover, many women referred to the study were not included, potentially causing a selection bias. Nonetheless our results provide insight in the effectiveness, acceptability and feasibility of the SAFER pregnancy intervention. Despite many efforts we failed to include women using alcohol. This might be due to the fact that most Dutch pregnant women who reported alcohol consumption in a recent questionnaire reported drinking only a few sips, and actual alcohol addiction might be less prevalent [11]. Another possibility might be that the stigma of alcohol consumption during pregnancy is more serious than that of smoking during pregnancy and women are less likely to admit alcohol consumption to their healthcare provider [44, 45]. Furthermore, during the study period the COVID-19 pandemic hit. Therefore, a number of group sessions was provided digitally. Perhaps participants deemed digital sessions less helpful as women who stopped smoking more often only attended live group sessions versus the women who did not stop smoking. Moreover, during the focus groups women expressed that online group sessions should not replace live group sessions. Secondly, biochemical validation of smoking cessation was temporarily halted and this might have resulted in participants falsely implying smoking cessation. Lastly, follow-up was short and therefore we were not able to study impact of the intervention on postpartum relapse. Concerning the focus group study, data analysis was performed by one researcher. To limit interpreter bias, the main focus group leader (VM) read the conclusions and agreed upon them.

Tappin et al. [46] conducted a randomised controlled trial (RCT) in the United Kingdom and provided pregnant women in the intervention group up to €446 for smoking cessation, in addition to routine care. Although the amount of money that could be earned was more than twice as much as in our intervention, the quit rate at 34 to 38 weeks of gestation, was fairly similar (23% vs 28% in our study). Comparison between the two studies is difficult due to various differences related to study design and intervention content. Many factors can explain possible differences, for example the addition of group sessions, the web-based platform and personal guidance. But it can also be hypothesised that it is not only the amount of incentives that motivates smoking cessation, but also the positive approach and the concrete goal that incentives, regardless of value, can facilitate..

Our findings indicate that an intervention combining group sessions, personal contact, an online platform and incentives is appropriate for women in need of extra support for smoking cessation before and during pregnancy [41]. Whilst this does not allow disentanglement of the individual impact of each intervention, the group sessions were highly valued by the participants. Most participants also regarded the incentives motivating to quit smoking, although fewer women actually believed it helped to quit smoking. This is in line with recent qualitative study among participants in an RCT which showed that smokers who received incentives more often quit compared to smokers not receiving incentives; nonetheless also in that study participants felt that the helpfulness of the incentives was low [47]. The web-based platform used in our study was found to be less appealing for this population, as most of our participants did not log on to the platform. Interestingly, the women who did log on the platform were more likely to have stopped smoking, although they rated the supportiveness of the platform neutral and expressed during the focus groups the platform not to be supportive in smoking cessation. To better support women in smoking cessation, a more personalised approach is possibly better valued. In this way, women can choose the support (e.g. an online platform or group sessions) that they need and prefer, decreasing the risk of dropout due to an unfit intervention. The CO breath test was found the be a concrete motivation for smoking cessation. This easy method can also be used by obstetric care providers to identify smokers and increase motivation to quit, as in some counties is already standard of care [35].

Recruitment was challenging, especially among women consuming alcohol. Previous research showed that 5% of Dutch women consume alcohol during pregnancy but that the majority of these women (89%) does not discuss this with their healthcare provider [12], impeding referral to an intervention. We concluded that the intervention is appealing and feasible for women who smoke during pregnancy or while planning pregnancy. Further tailoring of the SAFER intervention, e.g. by increasing the frequency of group sessions and offering more personalised support for specific problems (e.g. NRT or financial or housing aid) may increase its effectiveness. After these enhancements a (cluster) RCT could be the next step in studying effectivity of the innovated SAFER pregnancy intervention in supporting women in smoking cessation before and during pregnancy. However, it might be more cost-effective to study which elements of the intervention work for whom, so a more personalised approach is possible. Since the effectiveness of incentives for smoking cessation during pregnancy is already well-established [27, 28, 48–50], implementation should be pursued alongside additional study of more complex interventions [28, 51]. Moreover, as all women who successfully quit smoking were motivated to quit smoking before starting the study, more research on methods to motivate smoking cessation are needed to also properly support women who are less motivated to stop smoking.

## Conclusions

The SAFER pregnancy intervention seems to be a promising intervention, particularly for women with a low SES, to support smoking cessation before and during pregnancy. Almost a third of all participants (42% of those who finished the study) had successfully quit smoking at the end of pregnancy or after following six group sessions. A personal and positive approach was valued by the participants as an important prerequisite for success. The inclusion of participants was slow and early dropout was high (33%); these issues need addressing in further optimising of this intervention.

## Supplementary Information

**Additional file 1: Table S1.** Value of the incentives per occasion when smoking cessation was achieved.

**Additional file 2: Table S2.** Future perspectives on abstinence of women who quit smoking at the primary endpoint of the study*.

**Additional file 3: Figure S1.** Median appreciation of the contact with the researcher per questionnaire around group session^1^.

**Additional file 4: Table S3.** Sample demographics of the participants of the focus groups.

## Data Availability

The datasets generated and/or analysed during the current study are not publicly available due to identifiability, but are available from the corresponding author on reasonable request.

## Abbreviations

CI: Confidence interval
CO: Carbon monoxide
IQR: Interquartile range
MTSS: Motivation to Stop Scale
NRT: Nicotine replacement therapy
OR: Odds ratio
RCT: Randomised controlled trial
RR: Relative risk
SAFER: Smoke and Alcohol Free with EHealth and Rewards
SES: Socioeconomic status
