# Supplementary material for: Piloting a complex intervention to promote a tobacco and alcohol-free pregnancy: the Smoke and Alcohol Free with EHealth and Rewards (SAFER) pregnancy study
Source: BMC Pregnancy Childbirth. 2023 Jan 10;23:19. doi: 10.1186/s12884-022-05320-8 (PMC9830616; doi:10.1186/s12884-022-05320-8)
Supplement: Supplementary file 1 — Additional file 1: Table S1. Value of the incentives per occasion when smoking cessation was achieved. [file 12884_2022_5320_MOESM1_ESM.docx]

**Table S1 Value of the incentives per occasion when smoking cessation was achieved**

| **Consecutive number of times biochemically verified cessation*** | **Value of the incentive in euros if smoking cessation was achieved** |
| --- | --- |
| **1** | 15 |
| **2** | 20 |
| **3** | 20 |
| **4** | 30 |
| **5** | 40 |
| **6** | 60 |
| **Total** | **185** |

* During the COVID-19 pandemic biochemical validation was stopped and self-reported abstinence was used as the indicator of smoking cessation
