# Supplementary material for: Piloting a complex intervention to promote a tobacco and alcohol-free pregnancy: the Smoke and Alcohol Free with EHealth and Rewards (SAFER) pregnancy study
Source: BMC Pregnancy Childbirth. 2023 Jan 10;23:19. doi: 10.1186/s12884-022-05320-8 (PMC9830616; doi:10.1186/s12884-022-05320-8)
Supplement: Supplementary file 2 — Additional file 2: Table S2. Future perspectives on abstinence of women who quit smoking at the primary endpoint of the study*. [file 12884_2022_5320_MOESM2_ESM.docx]

**Table S2 Future perspectives on abstinence of women who quit smoking at the primary endpoint of the study***

| **Question** | **Answer** | **Pregnant**  **(n=9)** | **Not pregnant**  **(n=2)** | **Total**  **(n=11)** |
| --- | --- | --- | --- | --- |
| **Are you planning to remain abstinent?** | Yes | 5 (56) | 2 (100) | 7 (64) |
|  | In doubt, but I think so | 1 (11) | 0 (0) | 1 (9) |
|  | In doubt, but I do not think so | 0 (0) | 0 (0) | 0 (0) |
|  | No | 1 (11) | 0 (0) | 1 (9) |
|  | Missing | 2 (22) | 0 (0) | 2 (18) |
| **Do you believe you can remain abstinent** | Yes, absolutely sure | 1 (11) | 1 (50) | 2 (18) |
|  | Yes, pretty sure | 2 (22) | 1 (50) | 3 (27) |
|  | Yes, I think I can | 1 (11) | 0 (0) | 1 (9) |
|  | I doubt myself | 3 (33) | 0 (0) | 3 (27) |
|  | No, I do not think I can | 0 (0) | 0 (0) | 0 (0) |
|  | No, I am pretty sure I cannot | 0 (0) | 0 (0) | 0 (0) |
|  | No, I am absolutely sure I cannot | 0 (0) | 0 (0) | 0 (0) |
|  | Missing | 2 (22) | 0 (0) | 2 (18) |

* stated as n(%)
