# Supplementary material for: Piloting a complex intervention to promote a tobacco and alcohol-free pregnancy: the Smoke and Alcohol Free with EHealth and Rewards (SAFER) pregnancy study
Source: BMC Pregnancy Childbirth. 2023 Jan 10;23:19. doi: 10.1186/s12884-022-05320-8 (PMC9830616; doi:10.1186/s12884-022-05320-8)
Supplement: Supplementary file 3 — Additional file 3: Figure S1. Median appreciation of the contact with the researcher per questionnaire around group session1. [file 12884_2022_5320_MOESM3_ESM.docx]

**Figure S1 Median appreciation of the contact with the researcher per questionnaire around group session^1^**

^1^ Scores could range between 1 (highly disagree contact was helpful) and 5 (highly agree contact was helpful).
