# Supplementary material for: Piloting a complex intervention to promote a tobacco and alcohol-free pregnancy: the Smoke and Alcohol Free with EHealth and Rewards (SAFER) pregnancy study
Source: BMC Pregnancy Childbirth. 2023 Jan 10;23:19. doi: 10.1186/s12884-022-05320-8 (PMC9830616; doi:10.1186/s12884-022-05320-8)
Supplement: Supplementary file 4 — Additional file 4: Table S3. Sample demographics of the participants of the focus groups. [file 12884_2022_5320_MOESM4_ESM.docx]

**Table S3 Sample demographics of the participants of the focus groups**

| **Participants of the SAFER pregnancy study** | | | | | |
| --- | --- | --- | --- | --- | --- |
| **ID** | **Age (y)** | **Current smoking status** | **Smoking status partner** | **Smoking during intervention** |  |
| P1 | 32 | Quit | Yes | Successfully quit |  |
| P2 | 37 | Quit | Quit | Successfully quit |  |
| P3 | 26 | Quit | No partner | Successfully quit |  |
| P4 | 28 | Smoker | Yes | Quit and relapse |  |
| P5 | 30 | Smoker | Yes | Quit and relapse |  |
| P6 | 43 | Smoker | Never | During entire study |  |
| P7 | 32 | Quit | Yes | Successfully quit |  |
| **Leaders of the group sessions of the SAFER pregnancy study** | | | | | |
| **ID** | **Age (y)** | **Smoking status** | **Number of group sessions led** | **Profession** | **Experience in profession (y)** |
| L1 | 53 | Never | 8 | Smoking cessation coach | 5 |
| L2 | 45 | Quit | 3 | Mindfulness trainer | 5 |
| L3 | 42 | Quit | 5 | Lifestyle coach | 26 |
| **Healthcare providers who referred (potential) participants to the SAFER pregnancy study** | | | | | |
| **ID** | **Age (y)** | **Smoking status (yes/no)** | **Estimated number of patients referred** | **Profession** | **Experience in profession (y)** |
| H1 | 32 | No | 5-10 | Midwife | 8 |
| H2 | 50 | No | Approx. 10 | Midwife | 25 |
| H3 | 28 | No | 3 | Medical doctor | 3 |
| H4 | 31 | No | 3 | Medical doctor | 2 |
| H5 | 36 | No | Missing | Midwife | 13 |
| H6 | 56 | No | 5 | Gynaecologist | 20 |
| H7 | 32 | No | 5 | Midwife | 8 |
| H8 | 28 | No | 4 or 5 | Medical doctor | 2 |
